# Tackling the Growing Threat of Multidrug‐Resistant *Pseudomonas aeruginosa* in Sub‐Saharan Africa: Challenges, Strategies, and the Way Forward

**DOI:** 10.1155/ijm/6699909

**Published:** 2026-10-29

**Authors:** Barack Magwi Nyanswi, Ivy J. Mutai, Jael Obiero, Atunga Nyachieo, Anncarol Karanja

**Affiliations:** ^1^ Antimicrobial Resistance and Bacteriophage Biology Section Kenya Institute of Primate Research (KIPRE) Nairobi Kenya; ^2^ Health Program International Livestock Research Institute (ILRI) Nairobi Kenya ilri.org; ^3^ Becky Mayer Centre for Phage Research Division of Microbiology and Infection University of Leicester Leicester UK le.ac.uk; ^4^ Department of Biochemistry Microbiology & Biotechnology Kenyatta University Nairobi Kenya ku.ac.ke

**Keywords:** antimicrobial stewardship, healthcare strategies, multidrug resistance, *Pseudomonas aeruginosa*, Sub-Saharan Africa

## Abstract

*Pseudomonas aeruginosa* is a dangerous pathogen that has shown multidrug resistance in Sub‐Saharan Africa (SSA) and has significantly impacted healthcare, where poor infection control and limited resources are common. This narrative review examines strategies in the SSA region to control this pathogen, reports its prevalence, highlights key challenges, and suggests future research. Studies published between 2010 and 2025 in PubMed, Scopus, and Google Scholar, focusing on AMR surveillance, prevalence, infection control, and treatment strategies for AMR *P. aeruginosa* in SSA, were reviewed. Data were qualitatively and quantitatively summarized and mapped as descriptive median resistance (%) across SSA countries in a geographic heatmap. The results, illustrated in the heatmap, reveal substantial median resistance to common first‐line agents, whereas significant data gaps indicate a critical lack of standardized surveillance in the region. Nations such as South Africa and Kenya have progressed with surveillance and stewardship programs. However, challenges persist in other countries, especially in diagnostics, access to new antibiotics, and infection control. New emerging therapies, including phage therapy and new antibiotics, have shown promise but are limited by regulatory frameworks, costs, and availability. Infection prevention measures are equally weak, resulting in high infection rates due to overcrowding and inadequate hygiene facilities. A more integrated approach that strengthens surveillance, improves infection control, and increases access to novel treatments is needed to combat MDR *P. aeruginosa* in SSA. Future research must prioritize affordable diagnostics and bridge the “data silence” identified in the regional resistance mapping to address the region′s unique challenges.

## 1. Introduction

The worldwide use of antibiotics has led to an increase in instances of antimicrobial resistance (AMR), making it one of the most pressing global health threats of the 21st century [1]. AMR was directly responsible for roughly 1.27 million fatalities in 2019, with Western Sub‐Saharan Africa (SSA) showing the highest estimated AMR death rate of 27.3 deaths per 100,000 persons [2]. Structural determinants, such as limited diagnostic capacity, inadequate antimicrobial stewardship, weak surveillance systems, and constrained infection prevention infrastructure, have further amplified the regional spread of resistant pathogens [3, 4]. *Pseudomonas aeruginosa* is recognized by the World Health Organization (WHO) as a high‐priority pathogen due to its increasing AMR and substantial clinical burden [1]. As a member of the ESKAPE group of high‐risk pathogens, it exhibits diverse intrinsic and acquired resistance mechanisms that significantly limit therapeutic options [5].

To ensure clarity and comparability across studies, this review applies the international consensus definitions of multidrug‐resistant (MDR) and extensively drug‐resistant (XDR) phenotypes proposed by Magiorakos et al. (2012). MDR is defined as acquired nonsusceptibility to at least one agent in three or more antimicrobial categories, and XDR as nonsusceptibility to at least one agent in all but two or fewer categories [6]. These standardized definitions enhance comparability across studies in SSA, particularly in regions where inconsistent MDR classifications have been reported. Furthermore, this review acknowledges that there are disparate definitions of MDR in existing literature, for instance, defining MDR as resistance to more than one agent [7]. This necessitates a cautious interpretation of reported resistance percentages to ensure scientific accuracy in reporting *P. aeruginosa* MDR cases. MDR and XDR *P. aeruginosa* phenotypes, including carbapenem‐resistant *P. aeruginosa* (CRPA), defined here as resistance to at least one antipseudomonal carbapenem agent, continue to be increasingly documented worldwide and are associated with elevated mortality and limited treatment options [2, 8, 9]. In addition, the concept of difficult‐to‐treat resistance (DTR), defined as nonsusceptibility to high‐efficacy first‐line agents such as carbapenems, *β*‐lactam/*β*‐lactamase inhibitors, and fluoroquinolones, offers a clinically relevant framework for understanding therapeutic limitations in severe infections [10].

SSA has recorded some of the highest AMR‐related mortality rates globally, with Western SSA reporting higher numbers [2]. It is difficult to fully understand the true extent and effects of AMR in SSA because of the lack of reliable AMR data from the region, and several multinational analyses even exclude countries in the region due to insufficient reporting [2, 11]. Structural determinants, such as insufficient water, sanitation, and hygiene (WASH), poor waste management, hazardous food hygiene, inappropriate antimicrobial use in human and animal health, limited diagnostic capacity, and weak stewardship frameworks, play a significant role in the formation of AMR in SSA [3, 4, 11, 12]. Against this backdrop of constrained surveillance and fragmented data, the specific epidemiology, healthcare burden, and resistance patterns of *P. aeruginosa* remain poorly characterized. This review synthesizes current knowledge to provide a comprehensive overview of the pathogen′s regional impact, associated healthcare and socioeconomic challenges, and potential strategies for strengthening future AMR responses.

## 2. Methodology

This study is a narrative review conducted in accordance with the SANRA framework [13]. Relevant literature was identified through searches of PubMed, Scopus, and Google Scholar using combinations of terms related to *P. aeruginosa*, AMR, multidrug resistance, SSA, infection control, treatment, and antimicrobial stewardship. The search focused on English‐language research published between 2010 and 2025, with select landmark references included through early 2026. Literature identification was iterative and guided by relevance to the objectives and thematic areas of the review rather than by a predefined systematic search and screening protocol. The databases searched, principal search terms, and publication period are summarized in Table S2. For the regional epidemiological and quantitative resistance synthesis, studies reporting data from SSA were prioritized; relevant studies from outside SSA were also considered where they provided contextual evidence on resistance mechanisms, infection control, antimicrobial stewardship, or treatment. Primary research publications were prioritized for the review′s evidentiary content, whereas other relevant literature was considered where it provided contextual evidence for the thematic areas of the review. Data were extracted on study location, AMR surveillance procedures, infection control measures, treatment options, and results. A thematic analysis was conducted to identify significant strategies, obstacles, and trends. Findings across all included sources were grouped thematically into resistance mechanisms, regional epidemiology, clinical and therapeutic data, and infection control and stewardship strategies, allowing patterns and gaps across these domains to be identified and discussed narratively. Additionally, AMR data extracted from the studies were summarized to produce a regional overview of resistance. Thirty‐four studies reporting descriptive quantitative resistance or susceptibility data (i.e., reported percentages, not pooled or inferential estimates) were included in the country‐level summary underlying Table 1 and Figure 1.

**Figure 1:**
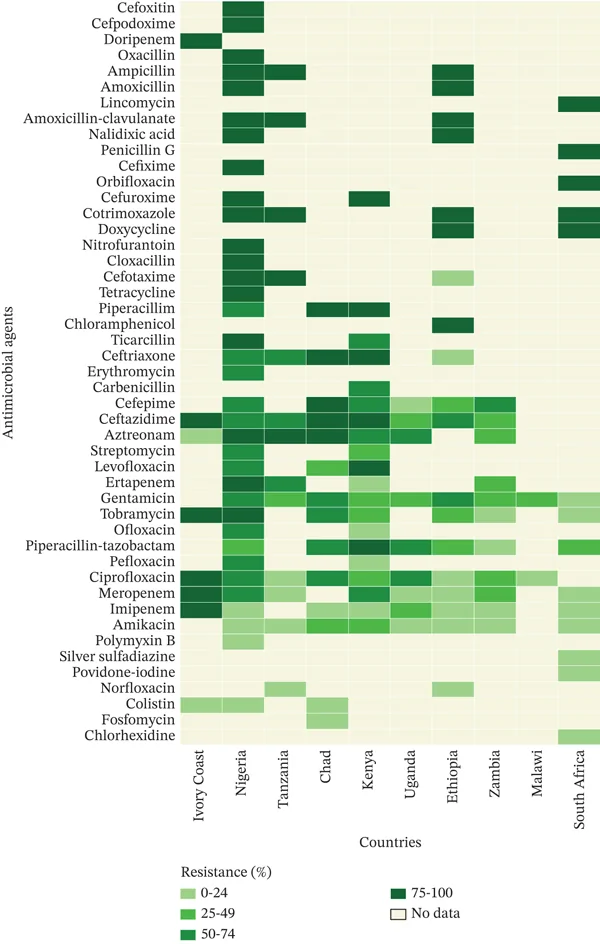
Heatmap showing median resistance (%) of antibiotics across 10 Sub‐Saharan African countries (of the 11 countries represented in Table 1, Ghana is not shown here as the source study did not report extractable quantitative resistance percentages for *P. aeruginosa*). Color scale ranges from light green (0% resistance) to dark green (100% resistance). Beige cells represent country–antibiotic combinations with no reported value used in the summary; values were not otherwise imputed. Only countries and antibiotics with reported data are presented. Resistance medians were calculated based on the unweighted resistance rates reported across included studies in each country, as a descriptive summary rather than a sample‐size–adjusted estimate; they should not be interpreted as inferential estimates. Where an individual antibiotic lacked study‐level data but the source study reported resistance for its broader antibiotic class, the class‐level value was applied to that agent; this was never used to override or combine with existing individual‐drug data. For countries represented by a single study, the value shown is that study′s observation rather than a calculated median. A total of 34 studies contributed to the 145 populated country–antibiotic values shown on this heatmap. Studies are summarized in Table 1, and the number of studies contributing to each country‐antibiotic value is provided in Table S1, which also identifies cells combining more than one isolate source category.

For each represented country, median resistance values were calculated from the resistance rates reported across studies and visualized as a heatmap in R to illustrate regional resistance patterns and data availability. The country‐level values shown in Figure 1 are descriptive, unweighted medians and are not intended as pooled or inferential estimates; accordingly, confidence intervals and effect‐size weighting were not applicable. Unweighted medians were selected because the included studies differed in sample size, study design, population, and source of isolates; the median was therefore used to summarize the reported study‐level resistance rates without allowing larger studies to disproportionately influence the country‐level summary. Across the 145 populated country–antibiotic combinations underlying Figure 1, the number of contributing studies ranged from one to nine, with 94 (65%) of these combinations derived from a single study. A full count of contributing studies for each country–antibiotic combination is provided in Table S1.

Where an individual antibiotic lacked study‐level data, but the source study reported resistance for its broader antibiotic class, the class‐level value was applied to that agent; this was never used to override or combine with existing individual‐drug data. Outbreak investigations and routine surveillance studies were retained to provide a broader descriptive evidence base of *P. aeruginosa* resistance in a region where such data remain scarce and, consistent with the descriptive rather than inferential aim of this synthesis, were not differentiated when calculating country‐level medians. Clinical, environmental, and veterinary studies were included to capture the distribution of *P. aeruginosa* and resistance across human, animal, and environmental settings; these sources were not treated as equivalent epidemiological populations. Breakpoint interpretation standards (e.g., CLSI or EUCAST) used in the source studies were not consistently reported, and variation in these standards should be considered when interpreting resistance rates across studies.

## 3. Summary of the Resistance Mechanism of *P. aeruginosa*

*P. aeruginosa* has developed a variety of antibiotic resistance mechanisms, which are classified into three categories: intrinsic resistance, acquired resistance, and adaptive resistance. Each of these methods enables the bacterium to evade the effects of antibiotics in unique ways, making it very difficult to treat (Figure 2).

**Figure 2:**
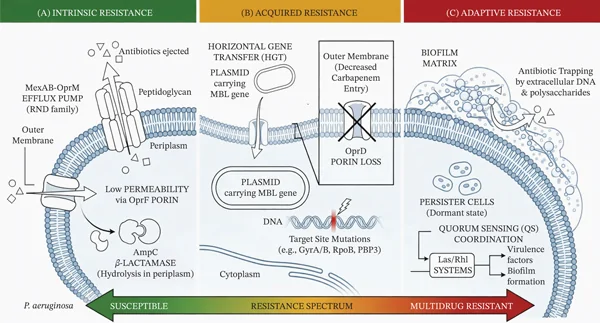
Multimodal resistance mechanisms of *P. aeruginosa*. The diagram illustrates the connection between three defense tiers: (A) Intrinsic resistance: constitutive defenses including the MexAB‐OprM efflux pump (active drug ejection) [14, 15], low‐permeability OprF porins (restricted entry) [16], and periplasmic AmpC\beta‐lactamase (enzymatic hydrolysis) [17, 18]. (B) Acquired resistance: resistance gained via horizontal gene transfer of MBL genes [19], loss of OprD porins (carbapenem resistance) [20], and chromosomal mutations in target sites (e.g., gyrA/B, PBP3) [21, 22]. (C) Adaptive resistance: population‐level survival strategies including biofilm matrix sequestration (eDNA/polysaccharides) [23–27], persister cell formation [23–25], and quorum sensing (Las/Rhl systems) coordination [28–30]. The figure represents a conceptual synthesis of established resistance mechanisms based on the relevant literature. Figure developed by the authors for this study; visual elements were refined using Microsoft Copilot with Adobe software.

### 3.1. Intrinsic Mechanism

*P. aeruginosa* possesses several intrinsic resistance mechanisms, including efflux systems, outer membrane impermeability, and antibiotic‐inactivating enzymes, which together allow it to withstand a wide range of antibiotics.

#### 3.1.1. The First Line of Defense: Efflux Systems

One of the most important mechanisms *P. aeruginosa* uses to evade antibiotics is its complex efflux systems. These transport systems, notably those from the Resistance Nodulation Division (*RND*) family, play an important role in antibiotic resistance. Efflux pumps work as bacterial “bouncers,” removing antibiotics and other potentially toxic substances from the cell. The MexXY system, which aggressively ejects a variety of medications, such as aminoglycosides, *β*‐lactams, macrolides, and fluoroquinolones, is a well‐researched example. Conversely, the MexAB–OprM system is well known for its capacity to confer resistance to several antibiotics, such as cephalosporins and ticarcillin, frequently in combination with other efflux systems [14, 15].

The adaptability of these efflux mechanisms presents a unique challenge for medical professionals who are treating the pathogen. When *P. aeruginosa* is exposed to antibiotics, it may upregulate the expression of genes encoding these pumps, further strengthening its defense. This flexibility means that even if some antibiotics work initially, treatment failure may still occur due to prolonged exposure, which increases efflux activity. Also, the *mexZ* gene strictly regulates MexXY, which ensures that the system is engaged only when necessary, and results in an efficient and resourceful resistance mechanism [31].

#### 3.1.2. The Fortress: Impermeability of the Outer Membrane

*P. aeruginosa* defends itself against antibiotics by active efflux as well as its outer membrane, a highly selective barrier that keeps many medications out of the cell. Lipopolysaccharides (LPS) and various porins, which are protein channels that regulate which molecules may pass through, make up the majority of this membrane′s complicated structure. Porins such as OprF (nonspecific) and OprD (specific to certain molecules) act as gatekeepers, restricting antibiotic access, whereas other gated porins, such as OprC, provide levels of protection [16].

Antibiotics have severe challenges in crossing this outer barrier. *β*‐lactams and quinolones use porin channels to enter cells, whereas aminoglycosides and polymyxins bind to LPS [32]. However, alterations in the outer membrane structure, such as LPS modification, might impair the antibiotics′ potency. Certain compounds, such as citric and lactic acids, can potentially change membrane permeability by altering LPS interactions, increasing the difficulty of treating *P. aeruginosa* infections [33].

#### 3.1.3. Enzyme Modification: Targeting Antibiotics at Their Source

*P. aeruginosa* employs more than just efflux and membrane defenses. It also aggressively modifies or destroys antibiotics using enzymatic mechanisms, making them ineffective. One well‐known example is the production of *β*‐lactamases, which hydrolyze the *β*‐lactam ring in drugs such as penicillins and cephalosporins. This class of enzymes is classified into four categories: A, B, C, and D, each having its own method for deactivating antibiotics. Class A, C, and D *β*‐lactamases break down *β*‐lactams using a serine‐based mechanism, but Class B enzymes, commonly known as metallo‐*β*‐lactamases (MBLs), require zinc for antibiotic breakdown [34].

The emergence of extended‐spectrum *β*‐lactamases (ESBLs), such as OXA‐type ESBLs, which may neutralize a broader range of *β*‐lactam antibiotics, is a growing concern. It is challenging to treat infections because of these enzymes′ resistance to aztreonam, cephalosporins, and penicillins [35]. *P. aeruginosa*′s defensive mechanism is further strengthened by its ability to manufacture AmpC *β*‐lactamase, an enzyme that confers resistance to aminopenicillins and cephalosporins. AmpC *β*‐lactamase is an inducible, chromosomally encoded class C enzyme that plays a central role in *β*‐lactam resistance in *P. aeruginosa* and is considered nearly ubiquitous among clinical isolates of this species [17, 18]. It can also be constitutively overexpressed due to regulatory mutations. Derepression via mutations in regulator genes (e.g., *ampD* and *dacB*) leads to hyperproduction, which may confer high‐level resistance to many *β*‐lactam antibiotics, including aminopenicillins, several cephalosporins, and even some carbapenems. In SSA, AmpC overproduction is a major contributor to *β*‐lactam resistance patterns observed in regional studies [36, 37].

In addition, MBLs such as Verona integron‐encoded metallo‐*β*‐lactamase (VIM), imipenemase (IMP), and New Delhi metallo‐*β*‐lactamase (NDM) are increasingly reported worldwide. Although the number of studies remains limited compared to other regions, MBL‐producing isolates have been documented in countries including Nigeria and Sudan and are frequently associated with high‐level carbapenem resistance [38, 39]. MBLs are of particular concern in many regions due to their ability to hydrolyze the *β*‐lactam ring of most *β*‐lactam antibiotics, including carbapenems. These zinc‐dependent enzymes hydrolyze most *β*‐lactams, except for aztreonam. This phenotypic “fingerprint” was notably observed in an Ivory Coast outbreak, where VIM‐2‐producing *P. aeruginosa* isolates exhibited 100% resistance to carbapenems while remaining universally susceptible to aztreonam [40]. However, aztreonam monotherapy is often clinically ineffective against MBL‐producing *P. aeruginosa* because most of these strains co‐express serine *β*‐lactamases (such as AmpC or ESBLs), which can hydrolyze monobactams [41]. Combining aztreonam with avibactam (or with ceftazidime/avibactam) is a promising strategy, as aztreonam evades MBL hydrolysis whereas avibactam inhibits co‐produced serine *β*‐lactamases such as ESBLs, KPC, and OXA‐48 [42]. This combination exhibits in vitro synergy, although the results are less consistent in *P. aeruginosa* than in *Enterobacterales*, and it is most effective when no additional resistance mechanisms, such as strong efflux pumps or porin mutations, are present [43].

*P. aeruginosa*′*s* defensive mechanism is further strengthened by its ability to manufacture enzymes that modify aminoglycoside drugs. Examples of these enzymes are aminoglycoside phosphotransferases (APHs), aminoglycoside acetyltransferases (AACs), and aminoglycoside nucleotidyltransferases (ANTs). These enzymes act by adding phosphate, acetyl, or adenylyl groups to antibiotic molecules, which effectively neutralizes drugs such as kanamycin, gentamicin, and tobramycin [44].

### 3.2. Adaptive Resistance in *P. aeruginosa*

*P. aeruginosa* has outstanding adaptive resistance mechanisms, including biofilm formation and quorum sensing (QS), which enable it to persist in harsh conditions, avoid the immune system, and resist drugs.

#### 3.2.1. A Protective Shield: Biofilm Formation

One of *P. aeruginosa*′s primary survival strategies is biofilm production. The mechanism involves bacteria adhering to surfaces and surrounding themselves with a protective matrix composed of polysaccharides, proteins, lipids, and extracellular DNA. The matrix provides structural support and acts as a barrier, preventing immune cells and drugs from reaching germs. This aspect is particularly relevant in conditions that favor biofilm persistence. Such conditions may include cystic fibrosis (where mucoid strains predominate), noncystic fibrosis bronchiectasis, chronic wounds, or the presence of medical devices (catheters or ventilators), which enhance tolerance via matrix protection, persister cells, and reduced antibiotic penetration [23–25]. Biofilms also aid in maintaining moisture, allowing bacteria to survive in dry conditions, storing nutrients to sustain growth, and demonstrating high enzymatic activity for resource utilization [45]. In chronic infections, such as those reported in cystic fibrosis patients, *P. aeruginosa* adopts a mucoid form that promotes biofilm formation, allowing the bacteria to survive in the lungs [46]. Furthermore, biofilms neutralize antibiotics by trapping them, as evidenced by studies showing that compounds such as kanamycin and tobramycin are sequestered before they can act, significantly reducing their efficacy [26, 27]. Efflux pumps contribute to resistance by actively removing drugs from bacterial cells, providing an additional layer of defense [47].

#### 3.2.2. QS: Communication for Survival

QS is another mechanism used by *P. aeruginosa* to survive the antibiotic effect. This bacterial communication system uses signaling molecules known as autoinducers. The signals help in coordinating collective behavior, such as biofilm formation and the production of virulence factors, which in turn enhance the ability of *P. aeruginosa* to infect its host [28]. The QS network in *P. aeruginosa* is highly complex due to the association of systems like the Las and Rhl, which work together to regulate important bacterial activities. The Las system triggers the production of virulence factors and biofilm components, whereas the Rhl system complements this by managing additional regulatory functions [29]. The systems ensure that the bacteria can adapt to their environment by changing between the free‐swimming (planktonic) state and biofilm formation when it is required to survive under different environmental conditions [30].

### 3.3. Acquired Resistance in *P. aeruginosa*

Acquired resistance in *P. aeruginosa* occurs through a variety of mechanisms, including mutations and horizontal gene transfer.

#### 3.3.1. Mutation

Mutation‐induced resistance is complicated and multidimensional, impacting a variety of important bacterial functions [48]. Mutations can overproduce enzymes such as *β*‐lactamases, which degrade *β*‐lactam antibiotics, rendering them ineffective. Mutations in *P. aeruginosa* boost the activity of efflux pumps like MexCD‐OprJ, which, in turn, remove antibiotics from bacterial cells efficiently, reducing treatment efficacy [49]. Overexpression of the chromosomal cephalosporinase ampC, which is driven by mutations in genes like *dacB* and *ampD*, is the major cause of *β*‐lactam resistance [50]. These mutations also activate regulatory systems such as BlrAB/CreBC, which enhance resistance in the bacteria [51]. Point mutations in AmpR, such as D135N and R154H, are significant players in *P. aeruginosa*′s resistance, as they further enhance the expression of *ampC* in MDR/XDR clones such as ST175 [52]. Numerous genes that are responsible for the production of amidases, PBPs, and lytic transglycosylases have been shown to influence *ampC* expression, further complicating the treatment of *P. aeruginosa* [53, 54].

Besides *β*‐lactam resistance, *P. aeruginosa* mutations affecting porin proteins, such as OprD, reduce membrane permeability, which contributes to resistance against carbapenem antibiotics like imipenem [20]. Efflux pump overexpression, caused by mutations in regulatory genes such as *mexR* and *nalB*, further lowers bacterial susceptibility to various antibiotics [41]. These mutations impact *β*‐lactams, fluoroquinolones, and aminoglycosides. Changes in DNA gyrase (*gyrA* and *gyrB*) and ribosomal proteins, which are antibiotic targets, tend to reduce the effectiveness of quinolones and aminoglycosides, respectively [21]. Alterations in lipid A of the bacterial cell wall, which can occur to resist polymyxin antibiotics, are enhanced specifically by the addition of L‐Ara4N [55]. Also, structural mutations in proteins like PBP3 enhance resistance against *β*‐lactams [5].

#### 3.3.2. Horizontal Gene Transfer

Besides mutation‐driven resistance, *P. aeruginosa* acquires AMR through a horizontal gene transfer mechanism (HGT) [56]. The process enables the bacterium to pick up resistance genes from other bacteria in the same environment through pathways such as transformation, transduction, and conjugation [57]. Many of these resistance genes are stored in mobile genetic elements such as plasmids, transposons, integrons, and prophages [58]. Integrons are particularly important in *P. aeruginosa* populations as they serve as repositories for resistance genes, especially genes conferring resistance to *β*‐lactams and aminoglycosides [59]. Genes encoding MBLs, such as Florence imipenemase (FIM), NDM, Germany imipenemase (GIM), Sao Paulo metallo‐beta‐lactamase (SPM), VIM, and IMP, are often transferred through integrons and plasmids, which enable the bacterium to resist a broad range of *β*‐lactam antibiotics [60]. Resistance genes in *P. aeruginosa* are often clustered within genomic resistance islands, rather than plasmids, although some plasmids play a significant role in spreading resistance [61]. IncP‐2 plasmids that were historically connected to tellurite and antibiotic resistance have been associated with the transfer of carbapenemase genes like *blaVIM-2*, *blaIMP-45*, and *blaIMP-9* [62]. The plasmids often carry class 1 integrons within transposons, which makes them key players in the rapid spread of resistance across bacterial populations [63].

## 4. Overview of Virulence Determinants of *P. aeruginosa*

*P. aeruginosa* employs a wide range of virulence determinants that contribute to host colonization, immune evasion, and tissue damage during infection. These factors act in a synergetic mode to enhance pathogenicity, especially in immunocompromised individuals [64]. The type III secretion system (T3SS) is one of the factors that enables the injection of effector toxins such as ExoS, ExoT, ExoU, and ExoY into host cells [65]. In the presence of these toxins, the host cell may experience disruption in its cytoskeletal organization, inhibit phagocytosis, and induce host cell death, which ultimately facilitates bacterial dissemination and immune evasion. *P. aeruginosa* produces phenazine pigments, especially pyocyanin (encoded by genes involved in the phenazine biosynthesis pathway such as *phzB1*), which is a redox‐active phenazine pigment that generates reactive oxygen species that end up interfering with host cellular respiration and lead to epithelial damage, inflammation, and impaired immune responses [66, 67]. The bacteria also produce elastase (LasB) and other extracellular proteases (alkaline protease [aprA]) that degrade host structural proteins such as elastin, collagen, immunoglobulins, and complement proteins, thereby promoting tissue invasion and immune system evasion [64]. Exotoxin A, which is encoded by the *toxA* gene, is another virulence factor that inhibits host protein synthesis through ADP‐ribosylation of elongation factor‐2, leading to host cell death by inducing tissue damage and systemic infections [68]. There are also motility structures that play an important role in colonization and infection. This motility is facilitated by flagella and type IV pili, which are encoded by genes such as *pilA* and *pilB*, and they also help in adhesion to host tissues and the initiation of biofilm formation [69]. This biofilm formation is further enhanced by alginate production (encoded by genes such as *algD*), which promotes the development of protective mucoid biofilms that shield bacterial cells from host immune responses and antibiotic exposure [70]. QS systems, including the Las, Rhl, and Pqs regulatory networks, may also induce expression of multiple virulence genes and biofilm formation in response to bacterial population density [24]. These virulence determinants may collectively work synergistically with AMR mechanisms to enhance the pathogenic potential of *P. aeruginosa*.

## 5. Prevalence Trends of Antimicrobial‐Resistant *P. aeruginosa* in the SSA Region

Epidemiological patterns of drug‐resistant *P. aeruginosa* in SSA are scarcely documented, making it an important subject for future research. Even available studies show marked heterogeneity in prevalence within the same country or between neighboring nations. CRPA rates, for example, range from 6.0% to 34.8% depending on the setting and study period [71]. This observed variability, together with the emergence of high‐risk clones and mobile genetic elements that carry *blaNDM-1*, *blaVIM*, *blaIMP*, and *blaOXA* variants, emphasizes the urgency for additional research into region‐specific trends to better understand the scope of resistance and guide future responses. Synthesis of available documentation has revealed studies spanning across various countries in the SSA region and including diverse patient populations (burns, wounds, bloodstream, respiratory, and environmental isolates), MDR/XDR rates, and phenotypic and genotypic resistance profiles, although heterogeneously covered, as shown in Table 1.

**Table 1:** Characteristics of studies reporting antimicrobial resistance profiles of *Pseudomonas aeruginosa* isolates in Sub‐Saharan Africa.

Study	Country	Patient population/isolate source category	Infection site/specimen type	MDR/XDR	High resistance rates (%)	High susceptibility rates (%)
Abdi et al., 2024 [1]	Ethiopia	Human clinical: 210 burn patients at Yekatit 12 hospital burn center	Burn wound swabs	33.3% MDR	GEN (63.0)	IPM (88.9); AMK (81.5); MEM (77.8); FEP (77.8); TZP (74.1); TOB (70.4); CIP (66.7); CAZ (59.3)
Godebo et al., 2013 [2]	Ethiopia	Human clinical: 322 patients with infected wounds	Various infected wounds: trauma (37.8%), abscess (29.8%), surgical incisions, burns, cellulitis	High MDR reported	AMP (97.3); DOX (83.8); SXT (87.9); CHL (74.3)	CIP (94.6); NOR (93.2); GEN (89.2); CRO (90.5); CTX (87.8)
Asamenew et al., 2023 [3]	Ethiopia	Human clinical: 348 patients with various clinical samples	Urine, blood, wound, sputum	45.9% MDR; 9.5% suspected XDR	GEN (62.2); CAZ (51.4); FEP (50.0)	CIP (85.1); IPM (71.6); AMK (70.3)
Deribie et al., 2014 [4]	Ethiopia	Human clinical: burn patients	Burn wounds	—	AMP (100.0); AMC (100.0); AMX (100.0); CHL (87.5); CAZ (100); NAL (95.0); DOX (97.5)	CRO (62.5); NOR (85.0)
Eliasi et al., 2020 [5]	South Africa	Veterinary: canine clinical cases (veterinary academic hospital)	Otitis externa, wounds/abscesses, urinary tract infections	High resistance reported (study used a broader, nonstandard definition of MDR)	LIN (98.0); PEN (96.0); ORB (90.0); TMP‐SMX (90.0); DOX (87.0)	IPM (94.0); TOB (88.0); AMK (84.0); GEN (82.0)
Jung et al., 2024 [6]	South Africa	Human clinical: patients with CRPA isolates; biobank of 82 samples from public/private labs	Respiratory sputum/endotracheal aspirate, pus/wound	XDR (all 3 blaNDM‐1 isolates resistant to all except colistin)	High resistance to carbapenems	—
Coetzee et al., 2013 [7]	South Africa	Human clinical: pediatric burn patients (2,632 admissions over 36 months)	Burn wounds	—	TZP (36.1)	TOB (96.7); MEM (93.4); GEN/AMK (91.8); CHX (100.0); SSD (86.8); PVP‐I (92.5)
75. Adenipekun et al., 2023 [8]	Nigeria	Human clinical	—	MDR reported	CXM (100.0); CAZ (100.0); AMC (95.7); CFM (93.6); ATM (97.9); CTX (91.5); CRO (91.5); LVX (91.5); FEP (95.7); GEN (87.2); MEM (61.7); OFX (61.7)	AMK (95.7); TZP (85.1); IPM (63.8)
Olalekan et al., 2023 [9]	Nigeria	Human clinical	Clinical samples	—	PIP (62.0); TZP (59.0); CAZ (52.0); FEP (45.0); GEN (59.0); TOB (55.0); CIP (71.0); LVX (74.0)	IPM (61.0); MEM (56.0)
Okafor et al., 2022 [10]	Nigeria	Environmental	Aquatic environments	MDR reported	CAZ (68.8); CIP (69.9); OFX (70.8); ETP (84.3); FEP (85.1); TIC (89.4); AMK (90.4); GEN (92.0); CTX (93.8); AMC (100.0); TOB (100.0); OXA (100.0); ATM (100.0)	IPM (60.6)
Awanye et al., 2022 [11]	Nigeria	Human clinical	Clinical specimens	MDR/XDR reported	FEP (52.9); ATM (67.3); TZP (72.1); CAZ (76.9)	IPM (91.3); PMB (84.6); GEN (78.8); LVX (75.0); CIP (68.3)
Smith et al., 2012 [12]	Nigeria	Human clinical	Surgical wounds	MDR reported	SXT (100.0); AMX (100.0); NAL (100.0); NIT (100.0); CXM (100.0); FOX (100.0); TET (95.0); AMC (95.0); OFX (80.0); GEN (80.0); CIP (70.0); IPM (60.0)	—
Adesoji et al., 2023 [13]	Nigeria	Environmental	Sewage	High MDR reported	AMC (82.5); AMP (75.0); CAZ (92.5); CXM (90.0); CIP (80.0); GEN (55.0); NIT (77.5); OFX (65.0)	—
Akinloye et al. [14]	Nigeria	Human clinical	Wound samples	All isolates MDR	MEM (100.0); FEP (100.0); TIC (100.0); AMC (100.0); CTX (100.0); AMP (100.0); CPD (100.0)	IPM (92.8)
Zubair and Iregbu, 2018 [15]	Nigeria	Human clinical	Clinical specimens	—	CAZ (51.0)	IPM (88.0); TZP (84.5); CST (84.0); FEP (73.0); AMK (69.5); GEN (66.5); CIP (67.5); MEM (71.9)
Olayinka et al. [16]	Nigeria	Human clinical: surgical unit patients	Clinical samples	MDR reported	OFX (82.6); PEF (58.7)	IPM (94.6); CIP (90.2); AMK (89.1); CAZ (78.3); GEN (64.2)
Akingbade et al. [17]	Nigeria	Human clinical	Wound samples	MDR reported	AMX (92.7); AMP (90.0); CLO (88.2); SXT (77.3); ERY (72.7); TET (70.9); STR (65.5); OFX (60.0)	CAZ (80.0); GEN (73.6); LVX (69.1); CRO (65.5); CIP (64.5)
Ettu et al., 2021 [18]	Nigeria	Human clinical	Clinical samples	—	Carbapenem resistance (20.5)	—
Kateete et al., 2016 [19]	Uganda	Human clinical: hospitalized patients at Mulago Hospital	Various clinical specimens	MDR reported	Low carbapenem resistance	Clinical: IPM (76.0); MEM (100.0); environmental: IPM (67.0); MEM (100.0)
Kateete et al., 2017 [20]	Uganda	Human clinical and environmental: hospitalized patients and environmental isolates at Mulago Hospital	Clinical and environmental samples	MDR reported	Clinical: CIP (78.0); TZP (56.0); CAZ (56.0); GEN (56.0); environmental: TZP (64.0)	Clinical: ATM (44.0); FEP (67.0); IPM (67.0); MEM (100.0); AMK (78.0); environmental: ATM (52.0); FEP (100.0); CAZ (58.0); IPM (79.0); MEM (100.0); AMK (88.0); GEN (61.0); CIP (61.0)
Mukomena et al., 2024 [21]	Zambia	Human clinical: hospitalized patients in Lusaka and Copperbelt provinces	Clinical samples from tertiary hospitals	MDR 73.3%; XDR 37.2%	CAZ (34.5); TZP (30.1); FEP (69.2); ATM (39.8); CIP (24.3)	AMK (87.3); TOB (86.4); GEN (79.6); IPM (58.3)
Yamba et al., 2023 [22]	Zambia	Human clinical: bloodstream infection patients at the referral hospital	Blood culture isolates	—	CAZ (29.0); CIP (64.0); Carbapenems (33.0)	—
Kaluba et al., 2021 [23]	Zambia	Human clinical: patients at a large tertiary referral hospital	Clinical samples	—	Very low resistance	IPM (94.0); TZP (95.2); AMK (91.0); CIP (69.0); GEN (63.1); CAZ (51.2)
Musila et al., 2021 [24]	Kenya	Human clinical: clinical isolates from Nairobi hospitals	Clinical samples	MDR reported	MEM (100.0); PIP (100.0); CAZ (100.0); FEP (93.0); LVX (100.0)	—
Kiyaga et al., 2022 [25]	Kenya	Human clinical: clinical isolates from six hospitals	Clinical samples	MDR 31%	PIP (79.0); LVX (70.0); TIC (57.0)	MEM (66.0); FEP (68.0)
Lord et al., 2021 [26]	Kenya	Human clinical	Urine, blood, wound samples	—	Aminoglycosides (42.1); fluoroquinolones (15.0)	Antipseudomonal carbapenems (100.0)
Mukaya et al., 2018 [27]	Kenya	Human clinical: Kenyatta National Hospital patients	Clinical specimens	MDR reported	TZP (96.0); CRO (82.0); CAZ (63.1); CXM (79.7); CAR (70.1); ATM (54.0); MEM (54.0)	CIP (65.7)
Jeannot et al., 2013 [28]	Ivory Coast	Human clinical: ICU patients	Bloodstream, urinary tract, pulmonary infection, and catheter contamination	MDR reported	IPM (100.0); MEM (100.0); DOR (100.0); CAZ (100.0); TOB (100.0); CIP (100.0)	ATM (100.0); COL (100.0)
Mushi et al., 2014 [29]	Tanzania	Human clinical: patients at Bugando Medical Centre (MDR gram‐negative isolates)	Clinical specimens: pus, blood, urine, aspirates	MDR reported	AMP (100.0); AMC (97.5); TMP‐SMX (92.7); CRO (73.2); CAZ (53.7); ETP (56.1)	CIP (82.9); GEN (68.3); MEM (80.5)
Mbare et al., 2023 [30]	Tanzania	Environmental	Environmental swabs and water samples from tertiary hospitals in Dar es Salaam	3 MDR isolates identified	ATM (88.4)	NOR (97.7); AMK (96.9); CIP (96.9)
Moremi et al., 2016 [31]	Tanzania	Human clinical: hospital patients at Bugando Medical Centre	Clinical specimens (blood, urine, pus, CSF, sputum)	—	Third‐generation cephalosporins (87.5)	AMK (90.9)
Codjoe et al., 2019 [32]	Ghana	Human clinical: patients with carbapenem‐resistant gram‐negative infections	Clinical specimens (mainly wound and urine) from four hospitals	—	—	—
Ahmat et al., 2023 [33]	Chad (N′Djamena)	Human clinical: hospitalized and nonhospitalized patients undergoing bacteriological examination	Urine and pus	—	CRO (100.0); CAZ (97.8); FEP (93.5); ATM (91.3); PIP (82.6); GEN (60.9); CIP (56.5); TOB (52.2); TZP (52.2); AMK (47.8); LVX (41.3)	FOS (97.8); CST (97.8); IPM (87.0)
Musicha et al., 2017 [34]	Malawi	Human clinical: adult and pediatric patients with suspected sepsis	Bloodstream infections (blood cultures)	—	GEN (33.6); CIP (12.8)	—

*Note:* Resistance rates are reported as percentages (%), with multidrug‐resistant (MDR) and extensively drug‐resistant (XDR) phenotypes highlighted where applicable. Dashes (—) indicate that no data were reported for that antibiotic–country pair in the reviewed literature.

Abbreviations: AMC = amoxicillin–clavulanate, AMK = amikacin, AMP = ampicillin, AMX = amoxicillin, ATM = aztreonam, CAR = carbenicillin, CAZ = ceftazidime, CFM = cefixime, CHL = chloramphenicol, CHX = chlorhexidine, CIP = ciprofloxacin, CLO = cloxacillin, COL = colistin, CPD = cefpodoxime, CRO = ceftriaxone, CTX = cefotaxime, CXM = cefuroxime, DOR = doripenem, DOX = doxycycline, ERY = erythromycin, ETP = ertapenem, FEP = cefepime, FOS = fosfomycin, FOX = cefoxitin, GEN = gentamicin, IPM = imipenem, LEV = levofloxacin, LIN = lincomycin, MEM = meropenem, NAL = nalidixic acid, NIT = nitrofurantoin, NOR = norfloxacin, OFX = ofloxacin, ORB = orbifloxacin, OXA = oxacillin, PEF = pefloxacin, PEN = penicillin G, PIP = piperacillin, PMB = polymyxin B, PVP‐I = povidone–iodine, SSD = silver sulfadiazine, STR = streptomycin, SXT = cotrimoxazole, TET = tetracycline, TIC = ticarcillin, TOB = tobramycin, TZP = piperacillin–tazobactam.

Cross‐country comparisons revealed several critical trends regarding current treatment regimens. These data were visualized as a heatmap to highlight regional hotspots and viable therapeutic windows, as seen in Figure 1.

Most notably, there is a high resistance to several older third‐generation cephalosporins and *β*‐lactams in multiple reported settings. The median resistance to ceftazidime, which historically is a frontline antipseudomonal agent, exceeded 50% in six of the eight countries reporting data, peaking at 97.8% in Chad and 81.6% in Kenya (Table 1). Similarly, high resistance to aztreonam was observed, with resistance reaching 97.9% in Nigeria and 91.3% in Chad, raising concerns regarding their reliability for empirical treatment in settings where comparable resistance patterns are observed. These findings should, however, be interpreted in the context of local antibiograms and isolate‐specific susceptibility testing rather than as evidence that these agents are uniformly unsuitable for empirical treatment across SSA. The high resistance to aztreonam may support further evaluation of combination therapies, including aztreonam + ceftazidime–avibactam, particularly in settings where MBL‐producing strains are prevalent. However, prospective clinical evidence from SSA evaluating the effectiveness of such strategies remains limited.

There is a lack of “class consistency” within the carbapenems. Many regions indicate that imipenem is relatively effective (South Africa 6.0% and Chad 13.0%), whereas meropenem shows much higher resistance in the same or neighboring countries (South Africa 93.4% and Nigeria 52.8%). This trend suggests that resistance is not just “carbapenem‐wide” and might be driven by various specific mechanisms, such as the loss of porin (OprD) [72], which specifically affects imipenem, or efflux pumps (MexAB‐OprM) that affect meropenem [73]. This observation means that clinicians cannot assume that if one carbapenem fails, the entire class is lost; rather, it highlights the importance of individual antimicrobial susceptibility testing and local antibiograms when selecting a carbapenem.

Despite high resistance in other classes, a few drugs remain associated with lower reported resistance in some settings. Amikacin median resistance is low in Tanzania (6.1%), Zambia (10.9%), and Nigeria (20.7%), showing how aminoglycosides are among the potential options for further evaluation in combination‐based treatment strategies where susceptibility is demonstrated. Amikacin resistance was generally lower than that observed for several other antimicrobial classes, although the South African veterinary study reported substantial resistance to agents such as lincomycin (98%), penicillin G (96%), orbifloxacin (90%), and doxycycline (87%), highlighting the importance of including animal‐associated isolates in One Health AMR surveillance. However, the lower resistance observed in selected countries should not be interpreted as evidence that amikacin should be prioritized empirically across SSA, as treatment selection must consider local susceptibility patterns, infection characteristics, patient‐specific factors, toxicity, and available clinical evidence.

Colistin resistance was relatively low in the limited country‐level data available, with median resistance of 16.0% in Nigeria, 2.2% in Chad, and 0% in Côte d′Ivoire. Nevertheless, colistin remains an important last‐line therapeutic option for infections caused by MDR/XDR *P. aeruginosa*, supporting continued susceptibility surveillance and antimicrobial stewardship to preserve its potential activity. Piperacillin–tazobactam shows a middle‐ground profile; while it is failing in Kenya (96.0%), it remains relatively effective in Zambia (17.5%) and Ethiopia (25.9%), indicating that its potential role as a carbapenem‐sparing option may vary substantially by setting and should be evaluated using local susceptibility data.

The observed patterns should be considered hypothesis‐generating for informing antimicrobial stewardship and laboratory surveillance, not treatment directives. For instance, the finding that the median ceftazidime resistance rate was above 50% in six of eight countries, alongside comparatively lower amikacin resistance in some settings, points toward ceftazidime‐sparing, amikacin‐containing strategies worth investigating where local data support them. The high aztreonam resistance and reported prevalence of MBL‐producing strains similarly justify evaluating newer combinations such as aztreonam–avibactam, through both laboratory surveillance and prospective clinical study [74]. None of this should be read as a regional prescription, and any treatment strategy should be guided by local antibiograms, individual isolate susceptibility results, patient‐specific factors, and applicable clinical guidelines.

## 6. Tissue Tropism, Clinical Presentation, Treatment Regimens, and Patient Outcomes

Different strains of *P. aeruginosa* exhibit clear tissue tropism, primarily manifesting in the respiratory tract, urinary system, bloodstream, and cutaneous wounds (Table 1). These tropisms are associated with clinical presentations such as sepsis, chronic wound infection, and ventilator‐associated pneumonia, often leading to variable and frequently poor outcomes [75]. A coordinated set of virulence factors has been shown to facilitate various tropisms; injection of cytotoxic effectors (ExoU, ExoS, ExoT, and ExoY) tends to disrupt cytoskeletal integrity, inhibit phagocytosis, and induce rapid necrosis or apoptosis, which end up facilitating dissemination, particularly in the lungs (pneumonia) and bloodstream (sepsis) [65, 76]. Host structural proteins (elastin, collagen, immunoglobulins, and complement) may be degraded by extracellular proteases (LasB elastase and alkaline protease AprA), which promote tissue invasion and necrosis in burn wounds and chronic skin or soft tissue infections [64, 77]. Motility structures promote initial adhesion to epithelial surfaces and medical devices (catheters and ventilators), and ROS generated by phenazines tend to damage respiratory epithelium and impair immune clearance, favoring chronic lung colonization [64, 78]. Together with other mechanisms, such as the production of alginate and QS systems, these mechanisms act synergistically to exploit compromised barriers (burns, catheters, and damaged lung epithelium) and establish infections with high morbidity in vulnerable hosts.

Most existing literature on AMR surveillance for *P. aeruginosa* in the SSA region focuses on microbiological characterization and resistance profiles, leaving a substantial gap in reporting patient‐level clinical information and the therapeutic regimens used. However, there are a few regional reports that give insight into these clinical complexities, such as strong tropism for extensive burns and extremities in Ethiopia, with infections often leading to prolonged hospitalizations that exceed 1 month [79]. Similarly, there is documentation of severe wound‐related complications in a large‐scale South African study, with challenges such as graft loss and the necessity for repeated surgical debridement [80]. To provide a broader context, several non–AMR‐focused studies have described similar patterns across Ethiopia, Kenya, South Africa, and other SSA countries, whereby postmortem data associate *P. aeruginosa* with various tropisms related to hospital‐acquired pneumonia deaths in pediatric cases [81]. This high burden of invasive and fatal *P. aeruginosa* infections in children is further illustrated by individual case reports, such as that of a child with a pan‐drug‐resistant *P. aeruginosa* strain isolated from an infected burn wound in Rwanda [77]. Despite a few cases reporting patients receiving inappropriate empirical therapy, particularly third‐generation cephalosporins, that is, in a Zambian study [82], there is a lack of granular data on treatment regimens and specific patient outcomes, which is an indication of the urgent need for integrated clinical‐microbiological research to better correlate resistance patterns with patient outcomes in this region.

## 7. Migration and Regional Dissemination of *P. aeruginosa*

Population mobility, including cross‐border travel, labor migration, displacement, and medical tourism, is a widely recognized driver of AMR dissemination [83] and has been documented in MDR *Enterobacterales* such as ESBL‐producing *Escherichia coli* and *Klebsiella pneumoniae* [84]. Whether a comparable pathway applies to *P. aeruginosa* in SSA remains a hypothesis rather than an established finding: No direct epidemiological evidence currently links population migration or medical travel to *P. aeruginosa* dissemination in the region, and any proposed transmission pathway is extrapolated from *Enterobacterales* data rather than pathogen‐specific evidence [85]. Existing SSA data on *P. aeruginosa* are fragmented and largely limited to localized outbreak reports, rather than the longitudinal genomic surveillance needed to track cross‐border spread of high‐risk clones. Testing this hypothesis would require multicenter molecular epidemiology studies integrating whole‐genome sequencing, patient travel histories, and longitudinal AMR monitoring [86, 87]. This approach has proven informative for tracking resistant lineages in other pathogens and settings. This gap in *P. aeruginosa*‐specific transmission data represents a clear priority for future AMR research in SSA, given the pathogen′s clinical significance in the region.

## 8. Current Strategies for Addressing MDR *P. aeruginosa* in the SSA Region

### 8.1. Surveillance, Infection Control, and Antimicrobial Stewardship

Monitoring AMR using effective surveillance systems is critical for tracking the spread of MDR organisms [88]. Many SSA nations are implementing the National Action Plan (NAP), a coordinated framework that emphasizes a One Health approach to addressing AMR [89]. However, implementation remains inconsistent. While all examined SSA nations have implemented NAPs, their efficacy varies greatly. South Africa and Kenya have made substantial progress with regular surveillance and stewardship programs [90, 91]. At the same time, Botswana and Namibia have yet to officially launch their NAPs, and Eswatini has only recently done so [91]. SSA efforts face challenges due to fragmented systems, primarily driven by inadequate resources, infrastructure constraints, and a lack of coordination among nations [92]. Countries like Kenya have made progress through policy measures, but they still face challenges due to fragmented implementation and the complexities of scaling sustainable methods [89]. Coordinated response activities are made more difficult, especially when defined data‐sharing mechanisms for AMR information are missing throughout the area, as demonstrated by studies conducted in Zambia, Tanzania, and Uganda [93]. Programs like the WHO′s Global Antimicrobial Resistance Surveillance System (GLASS) and the Fleming Fund have stepped in to provide vital external funding, bolstering data collection and reporting [94]. Sustainability remains an issue despite these initiatives [95, 96]. Many healthcare facilities are finding it difficult to maintain regular reporting once external financing ceases; they struggle with diagnostic testing due to inadequate infrastructure and lack the necessary consumables for routine surveillance [97, 98].

Infection prevention and control (IPC) techniques are critical for limiting the spread of MDR bacteria in hospital settings [93]. These include proper hand hygiene, environmental cleaning, and isolation of infectious patients. Unfortunately, many healthcare institutions in SSA face constraints, including overcrowded wards, poor sanitation, and a lack of essential IPC supplies [99, 100]. These settings are perfect for the spread of MDR infections such as *P. aeruginosa*. There is a noticeable difference in cleanliness between facilities. In Nigeria and Uganda, the high incidence of *P. aeruginosa* in hospital environments (86.4% and 33%, respectively) highlights major gaps in cleaning and infection control [101]. Similarly, in Kenya, poor WASH facilities contribute to a greater incidence of hospital‐acquired infections, with five of 14 hospitals rating below 60% on WASH performance [102]. While Ugandan healthcare personnel understand the importance of hand hygiene, they frequently struggle to put this knowledge into practice [103]. Building a good IPC culture involves more than just knowledge; it also requires strong leadership and ongoing mentoring to teach best practices. Waste management and documentation of IPC measures, identified as areas of concern in low‐ and middle‐income countries, exacerbate infection control issues across SSA [99, 103]. Improving IPC procedures, especially in resource‐constrained hospitals, is critical to lowering healthcare‐associated infections (HAIs).

### 8.2. New Therapeutic Approaches

To fight MDR *P. aeruginosa* in SSA, healthcare practitioners are increasingly using newer antibiotics such as ceftolozane–tazobactam and ceftazidime–avibactam [104, 105]. These drugs have demonstrated efficacy against a variety of resistant strains, particularly when used in conjunction with other therapies. Ceftolozane–tazobactam distinguishes itself by its ability to treat infections that standard antibiotics, such as ceftazidime, can no longer treat, as studies in SSA have demonstrated ceftazidime resistance patterns (Figure 1). Ceftolozane–tazobactam is an antibiotic combination that contains ceftolozane, a cephalosporin intended particularly to treat resistant Gram‐negative bacteria, and tazobactam, a beta‐lactamase inhibitor that prevents ceftolozane from being broken down by enzymes generated by resistant bacteria. Ceftazidime–avibactam is another promising combination that helps reduce resistance. Avibactam is also a beta‐lactamase inhibitor, allowing ceftazidime to retain its effectiveness against a broader range of resistant organisms. Although some strains of *P. aeruginosa* in Latin America have exhibited resistance to the ceftolozane–tazobactam combination [106], the drug remains significantly more effective than older, single‐agent cephalosporins for resistant infections. Ceftazidime–avibactam has demonstrated good results in trials from SSA, where more than 90% of *Enterobacterales* isolates from Cameroon, Ivory Coast, Nigeria, and South Africa were sensitive to this combination [107]. For *P. aeruginosa*, 88.9% of isolates were susceptible, demonstrating that this combination is highly effective against MDR strains in the region. These novel combination therapies offer a viable solution to the identified resistance patterns. Classic, single‐dose cephalosporins such as ceftazidime can provide a stronger, more dependable defense against AMR in SSA. The challenge lies in accessing these advanced antibiotics, as their high cost and limited availability in the region hinder widespread use [108]. The situation often leads healthcare facilities to rely on less effective treatments, further worsened by a lack of diagnostic tools needed to accurately identify resistance patterns.

Given the rising prevalence of MBLs in SSA, and the ability of these zinc‐dependent enzymes to hydrolyze nearly all *β*‐lactams (except monobactams), aztreonam has become a useful antipseudomonal agent [22, 109]. However, the ability of MBL‐producing *P. aeruginosa* to often co‐express serine *β*‐lactamases, including the ubiquitous chromosomal AmpC or acquired ESBLs, tends to compromise the clinical utility of this regimen by hydrolyzing aztreonam. Combining aztreonam with avibactam, either as aztreonam/avibactam or more commonly as aztreonam + ceftazidime/avibactam, tends to overcome this limitation [109]. Avibactam inhibits coproduced serine *β*‐lactamases, thereby protecting aztreonam from degradation while maintaining its activity against MBL‐producing pathogens. In vitro studies suggest that this strategy might restore activity against MBL‐producing *P. aeruginosa*, although clinical outcomes are less consistent than in *Enterobacterales* due to additional intrinsic resistance mechanisms [43]. Hence, further clinical evaluation is warranted in SSA to examine real‐world efficacy and optimal use. Phage therapy is emerging as a potential treatment option for MDR *P. aeruginosa* infections in SSA [110]. This technique offers a tailored treatment, as phages are highly selective for their bacterial hosts, allowing them to lyse *P. aeruginosa* cells while sparing beneficial microbiota, thereby preserving patient microbial diversity [111]. Across SSA, researchers have identified and sequenced phages specific to *P. aeruginosa*, including *Podoviridae* in South Africa, *Myoviridae* in Nigeria, and distinct phages in Côte d′Ivoire and Kenya [112–115]. In experiments conducted in Kenya, imported phage cocktails showed promising success in eliminating *P. aeruginosa* [116]. The clinical implementation of this strategy in SSA presents obstacles, including a shortage of clinical trial data and inadequate regulatory frameworks to allow therapeutic use [110]. Phage therapy has been successfully used in clinical trials for *P. aeruginosa* infections worldwide, indicating it is an achievable treatment option [117]. Establishing specialized phage banks and campaigning for regulatory support might help shorten the gap between laboratory research and clinical use [118, 119]. Such infrastructure would address the developing resistance challenge while reducing reliance on conventional antibiotics.

There is currently no vaccine for *P. aeruginosa* on the market, but recent research shows encouraging progress. OprF protein vaccination: Research has demonstrated that the highly conserved outer membrane protein OprF is a highly effective vaccination target. OprF vaccination improved survival rates and decreased bacterial burdens in critical organs after infection, according to studies [120]. OprF and a viral protein (VP22) were combined in a DNA vaccine that produced a significant antibody and cellular immunological response against *P. aeruginosa* [121]. IC43 and outer membrane vesicles (OMVs): IC43, which combines the proteins OprF and OprI, reached human trials but has shown little efficacy, possibly due to early infection in high‐risk intensive care unit patients [122]. Vaccines based on OMV continue to show promise as broad‐spectrum tools. OMVs containing PA0833 have been shown to elicit protective immune responses in mice, decreasing bacterial levels and increasing survival after infection [123].

Type III secretion system target (TSST): Another potential target is the PcrV protein, which is a part of the T3SS of *P. aeruginosa*. Mice treated with PcrV‐based vaccinations have shown significantly improved survival and decreased pro‐inflammatory markers following infection with the bacterium [124, 125]. Further research may bring these vaccines closer to practical use in high‐burden areas such as SSA. Bacterial extract‐based immunotherapies are another emerging avenue. Standardized formulations such as immunoprophylaxis with MV140 (Uromune), which is a polyvalent bacterial extract vaccine primarily targeting *Enterobacterales* for recurrent urinary tract infections, have demonstrated clinical benefit in preventing recurrent infections [126, 127]. These formulations can be tailored to include *P. aeruginosa* and aim to stimulate the innate and adaptive immune systems at the mucosal site of infection. Further research into both protein‐based vaccines and extract‐based immunotherapies may bring these interventions closer to practical use in high‐burden SSA areas.

## 9. Challenges Identified

### 9.1. Healthcare Infrastructure and Socioeconomic Barriers

Limitations in healthcare infrastructure and socioeconomic challenges may hinder the effective management of MDR infections in SSA [2]. Hospitals in remote and underprivileged areas may not always have the necessary diagnostic equipment to detect MDR strains on time [71]. This shortage causes treatment delays and can aid in the spread of MDR infections, ultimately increasing patient morbidity [88]. Countries face severe limitations in both infrastructure and the number of sufficiently qualified healthcare professionals to undertake specialized tests, limiting diagnostic capacity and accurate management of MDR illnesses [103, 128]. Economic constraints may add another layer of difficulty in the fight against AMR [98]. In Uganda, diagnostics and effective treatment options are costly, placing a financial strain on both the healthcare system and patients. Many patients cannot afford the recommended regimens due to the costs [129]. This economic burden often forces patients to abandon treatment early and, in many cases, to resort to lower‐quality alternatives, thereby increasing AMR [108]. In Ghana, research highlights that limited access to quality healthcare contributes to the spread of resistant strains, as socio‐economic factors and infrastructural limitations interact to increase the region′s AMR challenges [130].

### 9.2. Regulatory and Policy Gaps

The lack of elaborate regulatory frameworks governing antibiotic prescription and use contributes to the rise of MDR illnesses in SSA [71]. Many nations in the region have limited controls on over‐the‐counter antibiotic sales, making antibiotics widely available to the public without professional oversight, which frequently leads to misuse and abuse [131]. According to studies, unregulated access to antibiotics is a major cause of AMR, as it frequently leads to subtherapeutic doses and self‐medication behaviors, which fuel resistance [132]. Weak infection control practices in healthcare settings also contribute significantly [88]. Insufficient implementation of hospital cleanliness standards, combined with limited sterilization resources, considerably increases the risk of HAIs caused by resistant strains, particularly in high‐turnover institutions. A growing yet often overlooked policy challenge is the role of population mobility and medical tourism in the regional dissemination of MDR strains. While the risk of colonization with MDR Gram‐negative organisms is frequently discussed in relation to *Enterobacterales*, it is equally relevant for *P. aeruginosa*, especially in African countries serving as regional medical hubs [85]. The lack of standardized cross‐border screening and referral protocols allows resistant clones to “hitchhike” across national boundaries, complicating local containment efforts [83, 133]. The absence of standardized national policies to enforce strict infection control and monitor antibiotic prescriptions adds to the spread of MDR *P. aeruginosa*, emphasizing the need for regulatory measures to successfully manage resistance [134]. Additionally, restrictions in terms of authorization for the clinical use of phage therapy are significant in the SSA region, making it difficult even to give the phages as a type of treatment, unless for compassionate use [110].

## 10. Implications for Future Practices and Directions

Addressing MDR infections needs a multifaceted approach that reinforces clinical practices, emphasizes research, and supports public health activities. The first step is to improve infection control in healthcare settings [135]. Kenya′s recent improvements in hospital cleanliness standards have significantly reduced HAIs, indicating a model that could be replicated in high‐traffic hospitals across the region to combat the spread of resistant Pseudomonas strains [89, 102]. Upgrading diagnostic capability is also critical [136, 137]. In resource‐limited areas like Uganda, establishing affordable, rapid diagnostic centers would help identify resistant infections early, reducing transmission and enhancing patient outcomes [93, 129]. Creating national AMR monitoring strategies in SSA could improve antibiotic management, diagnostics, and resistance reporting [138]. Actively exchanging surveillance data would help to assist decision‐making and policy development [139].

Tailoring research to establish treatment guidelines that are specific to particular regions is also crucial, particularly in areas where antibiotic misuse is common due to unregulated over‐the‐counter availability, such as in Nigeria and Ghana [130, 131]. Alternative treatments, such as phage therapy, have shown promise as a complement to standard antibiotics and should be employed [110, 118, 119]. Countries can also form public–private partnerships (PPPs) and follow successful worldwide models; these collaborations help accelerate AMR efforts in SSA by securing critical research money and resources [140, 141]. Public health initiatives centered on community education are equally vital, and examples such as Rwanda′s initiative to educate healthcare students demonstrate how raising awareness about responsible antibiotic use can reduce AMR incidence [142]. Finally, regional and global collaboration in AMR research is critical for sharing experience and resources [138]. Cross‐border activities, particularly those backed by the WHO, lay the groundwork for a coordinated response and promote shared solutions to AMR, benefiting the entire SSA [143]. Furthermore, surveillance strategies must be expanded to include molecular tracking of high‐risk clones across migration corridors [87]. Establishing regional genomic surveillance is essential to identify whether the emergence of MDR *P. aeruginosa* in a specific locale is due to indigenous evolution or the introduction of exogenous strains through medical travel and regional migration. Adopting a One Health approach is critical in addressing AMR from human, animal, and environmental perspectives [144, 145]. This integrated paradigm promotes cross‐disciplinary collaboration, hence improving AMR interventions and public health outcomes in SSA [89].

## 11. Limitations

This review has several limitations. First, as a narrative rather than systematic review, study selection was guided by relevance and author judgment rather than an exhaustive, reproducible protocol. The search was also restricted to English‐language publications, which may exclude relevant regional data published in French or Portuguese, which is a meaningful gap given the number of Francophone and Lusophone countries in SSA. Second, the studies summarized in Table 1 also varied considerably in design, sample size, and population source, with clinical, environmental, and veterinary isolates presented within the same summary table; this heterogeneity limits direct comparability across settings. For four countries (Nigeria, Uganda, South Africa, and Tanzania), 29 of the 145 populated heatmap cells combine data from more than one isolate source within a single median, identified in Table S1; source‐stratified subgroup summaries were not produced, as most of these cells derive from only two contributing studies, and splitting them further would yield single‐observation values rather than more robust descriptive estimates. The number of studies contributing to individual country–antibiotic estimates also varied, and estimates based on a single study or a small number of studies may therefore reflect the characteristics of those specific study populations rather than broader country‐level resistance patterns. Third, the breakpoint interpretation standards (e.g., CLSI or EUCAST) used by individual source studies were not systematically extracted during data collection; where this varies across studies, it may affect the comparability of reported resistance percentages. Fourth, the median values underlying Figure 1 are unweighted descriptive statistics rather than pooled estimates; for countries represented by a single study (Chad, Malawi, and Ivory Coast), the reported value is that single study′s observation rather than a true median. For example, the Ivory Coast value in particular derives from an ICU outbreak investigation (*n* = 12) [40] and should not be interpreted as representative of community‐level resistance in that country; the same limitation would apply to any single‐study country value drawn from a similarly narrow clinical context. The inclusion of outbreak, routine surveillance, environmental, and veterinary studies broadens the descriptive evidence base but introduces differences in sampling frames and epidemiological context that limit direct comparison between studies and countries. Finally, patient‐level clinical outcome and treatment‐response data were sparse across the studies reviewed, and direct evidence on the role of migration in regional *P. aeruginosa* dissemination remains limited. As the volume of available *P. aeruginosa* surveillance data in SSA grows, future studies, including systematic reviews with predefined study‐selection criteria, standardized extraction of susceptibility‐testing standards, and stratification by population, study setting, and isolate source (clinical, environmental, and veterinary), could improve the comparability of the evidence. Where sufficient homogeneous data are available, meta‐analytic approaches could provide more comparable pooled estimates, whereas prospective clinical studies would strengthen evidence on treatment outcomes.

## 12. Conclusion

A united effort that combines strong infection control, antimicrobial stewardship, and innovative treatments is needed to address the threat of MDR *P. aeruginosa* in SSA. Some countries have made progress with surveillance and national action plans, but many are still facing major challenges, including limited resources, poor hygiene infrastructure, and inconsistent implementation. For example, the struggles of healthcare facilities with overcrowding and a lack of essential WASH services are creating ideal conditions for the spread of resistance. New antibiotics like ceftolozane–tazobactam offer promise, but their high costs and limited availability prevent their widespread use in the region. A potential solution, such as the alternative use of phage therapy, has emerged, but more research and stronger regulatory support are needed to make it a viable option. The key to fighting resistance, in the long run, should be strengthening diagnostic tools and improving regional collaboration. Ultimately, the solution lies in a One Health approach that integrates efforts across human, animal, and environmental health. In addition, better infection control practices, antimicrobial stewardship, and improved WASH facilities are key factors in the fight against resistance. We can begin to combat the rising threat of MDR *P. aeruginosa* by addressing the issues outlined above, thereby improving health outcomes across SSA.

## Author Contributions

The authors affirm their contributions to the paper as outlined below: B.M.N.: conceptualization, methodology, investigation, data curation, formal analysis, visualization, writing – original draft. A.K.: conceptualization, supervision, writing – review and editing. I.J.M.: formal analysis, writing – review and editing. J.O.: writing – review and editing. A.N.: formal analysis, supervision, validation, writing – review and editing.

## Funding

No funding was received for this manuscript.

## Disclosure

All authors approved the final version of the manuscript.

## Conflicts of Interest

The authors declare no conflicts of interest.

## Supporting Information

Additional supporting information can be found online in the Supporting Information section.

## Supporting information


**Supporting Information 1** Table S1 (Excel workbook) reports the number of studies contributing to each of the 145 populated country–antibiotic combinations underlying Figure 1. It contains three sheets: (S1a) the list of contributing studies by country and antibiotic, (S1b) a country‐by‐antibiotic matrix of study counts, and (S1c) the resistance values used.

**Supporting Information 2** Table S2 (Excel workbook) summarizes the databases searched.

## Data Availability

No new datasets were generated; all data were derived from published studies and are presented within the manuscript.
